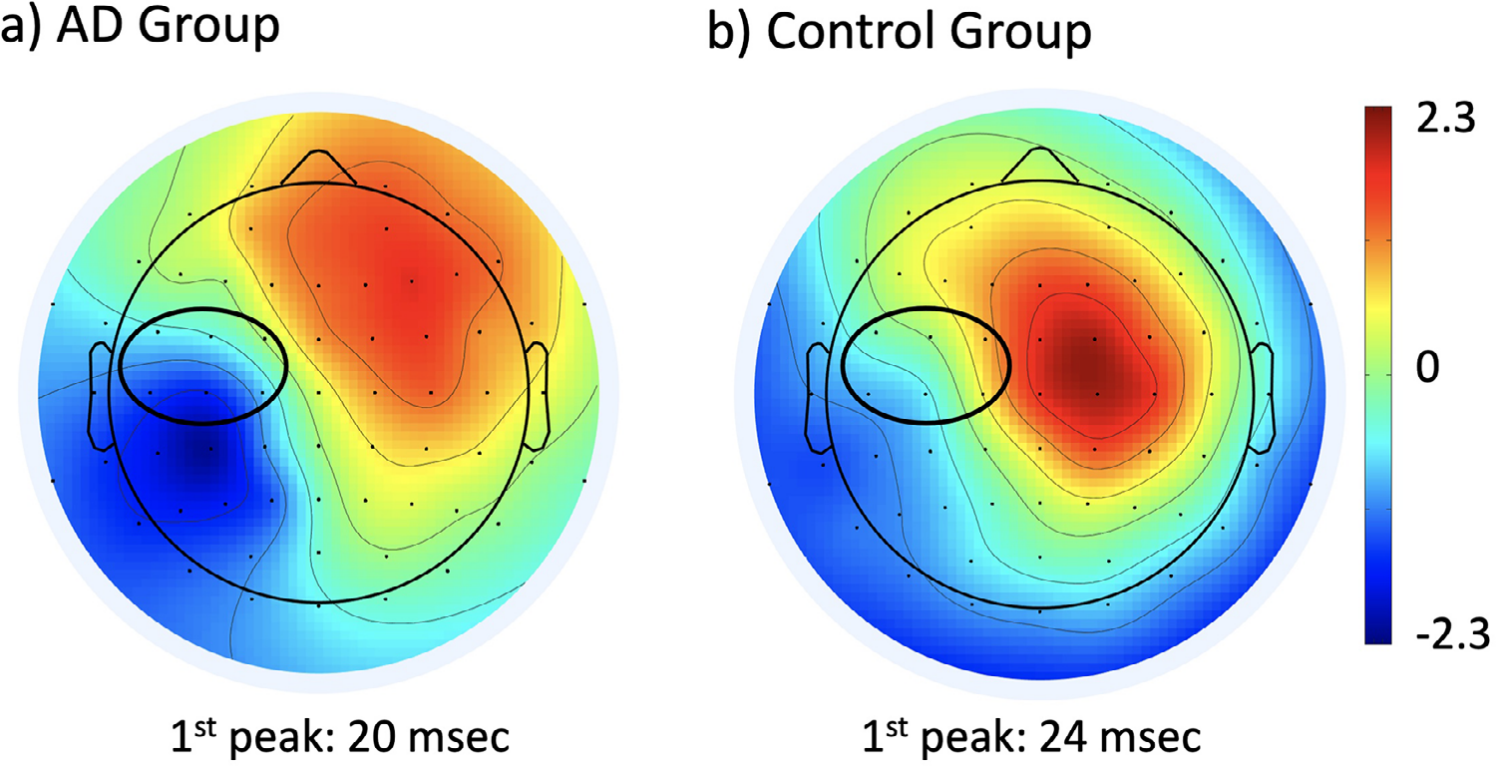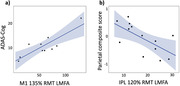# TMS‐EEG excitability measures are related to global and local cognitive function in early symptomatic AD

**DOI:** 10.1002/alz.088414

**Published:** 2025-01-09

**Authors:** Stephanie S. Buss, Recep Ozdemir, Brice Passera, Peter J. Fried, Daniel Manning, Isabelle Woods, Grace Desmond, Lynn Shaughnessy, Daniel Z. Press, Mouhsin Shafi

**Affiliations:** ^1^ Berenson‐Allen Center for Noninvasive Brain Stimulation, Beth Israel Deaconess Medical Center and Harvard Medical School, Boston, MA USA; ^2^ Department of Neurology, Harvard Medical School, Boston, MA USA; ^3^ Berenson‐Allen Center for Noninvasive Brain Stimulation, Beth Israel Deaconess Medical Center and Harvard Medical School, Boston, MA, USA, Boston, MA USA

## Abstract

**Background:**

Cortical excitability is elevated in Alzheimer’s disease (AD). Transcranial magnetic stimulation‐evoked responses on electromyography (EMG) and electroencephalography (EEG) have captured this increased excitability in motor brain regions. However, it is not yet known if increased excitability is also present in the parietal lobe or the extent to which excitability is related to cognition.

**Method:**

TMS‐EEG data from 29 participants with biomarker‐confirmed AD (CDR 0.5‐1, age 57‐81, 45% female) and 38 cognitively unimpaired older controls (CDR 0, age 57‐89, 59% female) were analyzed. Single‐pulse TMS was applied to left motor cortex (M1) and inferior parietal lobule (IPL) at 120% of resting motor threshold (RMT) and 135% of RMT (in 12 AD and 22 controls). The early (15‐40 msec) TMS‐evoked local mean field amplitude (LMFA) was assessed from electrodes near each TMS target. Group differences in LMFA were assessed separately for each site and intensity, controlling for age, sex, and scalp‐to‐cortex distance. A subset of AD participants had cognitive testing scores available for the Assessment Scale‐Cognitive subscale (ADAS‐Cog, n=10) and a Parietal Composite Score (averaged z‐scores of Benton Judgement of Line Orientation, ADAS‐Cog Maze 3 time, and WRAT 4 Math, n=13). Cognitive scores were related to LMFA at each site and intensity using separate linear models.

**Result:**

In M1, 135% RMT evoked a larger LMFA in AD than in older controls (R^2^adj=0.17, p=0.036). Visual inspection of the M1 135% RMT evoked responses also revealed higher local responses in AD (Figure 1). There were no between‐group differences for other conditions (p‐values > 0.100). In AD, higher M1 LMFA at 135% RMT was related to worse global cognition on the ADAS‐Cog (R^2^adj=0.68, p=0.002, Figure 2a), but not the Parietal Composite Score. Higher IPL LMFA at 120% RMT was related to worse performance on the Parietal Composite Score (R^2^adj =0.38, p=0.015, Figure 2b), but not the ADAS‐Cog.

**Conclusion:**

TMS‐EEG reveals elevated motor‐area excitability in AD. Excitability measures showed a double‐dissociation with cognition: motor excitability is related to global cognition and parietal excitability is related to parietal function. TMS‐EEG may be useful to measure target engagement for future therapies seeking to restore normal neuronal excitability in AD.